# Effects of Car Painting Vapors on Spirometric Parameters in Automobile Painting Workers

**Published:** 2019-04

**Authors:** Maryam Saraei, Gholamreza Pouryaghoub, Sahar Eftekhari

**Affiliations:** Center for Research on Occupational Diseases (CROD), Tehran University of Medical Sciences (TUMS), Tehran, Iran.

**Keywords:** Automobile painting, Spirometric parameters, Solvent-based paints

## Abstract

**Background:**

Automobile spray painting is considered a high-risk occupation for respiratory diseases. The present survey aimed to assess the effects of automobile paint vapors on spirometric parameters among workers of a painting workshop in a large automobile manufacturing plant in Iran.

**Materials and Methods:**

This cross-sectional study was conducted on 820 workers of an automobile manufacturing plant, including 431 spray painters (case group) and 389 assembly line workers (control group). Spirometry was conducted for all participants under standard conditions, according to the American Thoracic Society (ATS) Clinical Practice Guidelines. The forced expiratory volume in one second (FEV1), forced vital capacity (FVC), FEV1/FVC, and forced expiratory flow at 25% and 75% of the pulmonary volume (FEF25-75) were reported.

**Results:**

Painters with more than ten years of work experience had significantly lower predicted values of FEV1/FVC (P= 0.005), FEV1 (P=0.008), and FEF25-75 (P=0.003), compared to the control group. Also, painters who were exposed to solvent-based paints were not significantly different from those exposed to water-based paints in terms of spirometric parameters (P>0.05).

**Conclusion:**

The results indicated the impact of automobile spray painting on the spirometric parameters. A slight decrease in the mean values of these parameters calls for attention to occupational safety, regular medical examinations, and effective measures.

## INTRODUCTION

Exposure to different respiratory hazards in the workplace can lead to short- and long-term complications on the pulmonary function test (PFT). Chronic respiratory diseases represent a public health challenge in both developed and developing countries (1–3). Automobile painting is classified as a high-risk occupation for respiratory disorders and asthma, according to the European Community Respiratory Health Survey (ECRHS) (4).

Diisocyanates are compounds used in the production of polyurethane foams, adhesives, and paints (5). Generally, paint is a common source of isocyanate exposure (6). In automobile body painting workshops, exposure to diisocyanates occurs through the respiratory tract and the skin, with possible impacts on the respiratory system in various ways, such as irritation, asthma development, hypersensitivity pneumonitis, and asymptomatic acceleration of the pulmonary function (7,8). The risk of respiratory disorders is a function of multiple factors, such as the concentration of paint vapor, formulation or composition of the paint (oil-based or water-based), and duration of exposure (9).

Overall, vehicle spray painters have shown lower pulmonary function indices (10, 11). Despite the well-known risks of exposure to diisocyanates, the manufacture and application of these compounds are still increasing (12). Monitoring of exposure to these chemicals during automobile body painting, implementing occupational hygiene and establishing medical surveillance programs (e.g., periodic PFTs for exposed workers) play essential roles in identifying the affected workers and decreasing the risk of respiratory disorders. Generally, PFTs are robust tests for detecting the effect of certain chemical exposures on the pulmonary function.

With this background in mind, in this study, we aimed to examine the potential effects of exposure to permissible levels of isocyanate on spirometric parameters in automobile body painters.

## MATERIALS AND METHODS

### Study population

**Figure 1.**
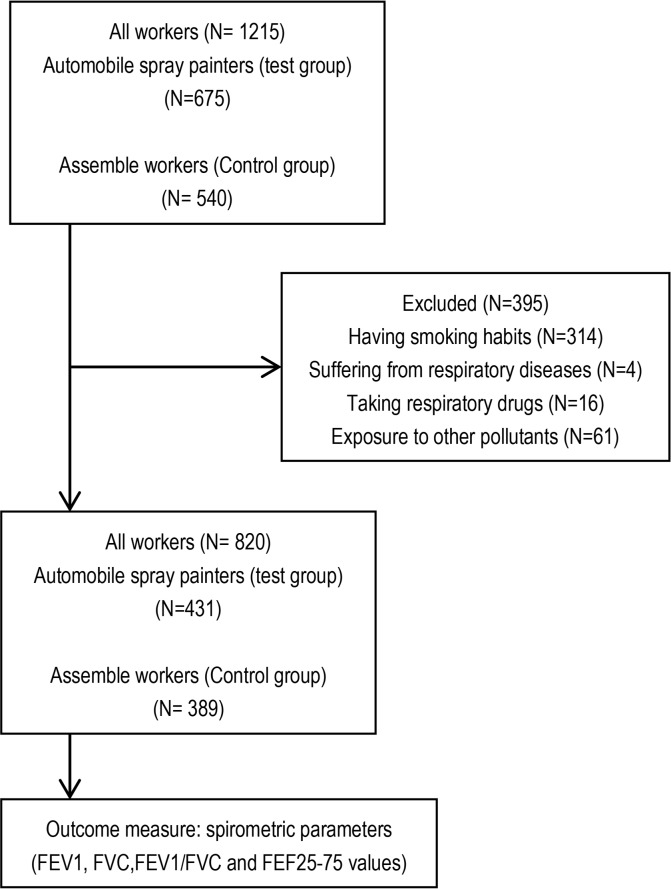
Project Outline

This analytical cross-sectional study consisted of 431 automobile spray painters aged 27–54 years (case group), as well as 389 workers in the same age group (control group), who were randomly selected from the assembly room of one of the largest automobile manufacturing plants in Iran. This study was conducted from March 2015 to November 2016. The exclusion criteria were as follows: 1) smoking; 2) respiratory disorders, such as pneumonia, asthma, and bronchitis; 3) consumption of respiratory drugs; and 4) exposure to other pollutants at home or in the workplace (Figure 1). Also, workers with less than five years of work experience were excluded from the study. All participants were healthy with normal physical examination results.

A self-made questionnaire was used to extract the subjects’ information regarding smoking habits, work experience, and the presence of diseases. The patients’ height and weight were measured using standard instruments. The body mass index (BMI) was also calculated as weight/height^2^ (kg/m^2^). Painters were divided into two groups, based on exposure to solvent- or water-based paints. The Research Ethics Committee of Tehran University of Medical Sciences approved the study.

### Spirometry

A trained expert physician performed spirometry using the MIR Spirobank^®^ spirometer manufactured by MIR Cinematografica Company in Italy according to the American Thoracic Society (ATS) Clinical Practice Guidelines (13). The PFTs were carried out in the morning hours, and the participants were given verbal explanations about the reliability of the test. A minimum of three acceptable forced vital capacity (FVC) maneuvers was performed in the sitting position with nose clips. The best maneuver with the optimal curve was selected for further analysis. Spirometric parameters, including forced expiratory volume in one second (FEV1), FVC, FEV1/FVC, and forced expiratory flow at 25% and 75% of the pulmonary volume (FEF25-75), were reported.

### Statistical analysis

SPSS Version 22 (IBM, USA) was used for statistical analysis. The normal distribution of variables was examined using the Kolmogorov-Smirnov test. Independent samples t-test and its non-parametric equivalent (Mann-Whitney U test) were used to compare the spirometric parameters between the groups. P<0.05 was considered statistically significant for all tests.

### Ethical Considerations

All participants signed the written informed consent forms. Participation in this study was voluntary, and the patients’ information remained confidential through anonymous data collection. The Ethics Committee of Iranian Ministry of Health Research in Tehran approved the study protocol (Project No.: 1395-587).

## RESULTS

The study population consisted of 820 monoracial male workers, aged 27–54 years (mean: 36.73, SD: 3.70). No significant difference was observed regarding the mean age of exposed and non-exposed workers (36.73±2.98 vs. 36.74±4.36) (P=0.96). The mean work experience of exposed workers was significantly higher than that of non-exposed workers (13.01±3.15 vs. 12.30±4.04), which was clinically insignificant. Also, there was no significant difference in the mean height and weight of the two study groups (175.92±6.30 vs. 175.28±6.33 and 82.31±11.83 vs. 81.86±12.84). The predicted values were used to compare each parameter between the groups after controlling for the effects of age and height on the spirometric parameters. The results are demonstrated in Table 1.

**Table 1.** comparing spirometry indexes between painters and control group

**Study Factors**	**Exposed Group**	**Unexposed Group**	**P value**
**All Participants**	**N= 431 (52.6%)**	**N= 389 (47.4%)**	
**FEV1/FVC** *(%)*	78.62±5.54	79.56±4.73	0.009^*^
**FVC to predicted ratio** *(%)*	0.94±0.10	0.95±0.17	0.59
**FEV1 to predicted ratio** *(%)*	0.92±0.11	0.94±0.15	0.02^*^
**FEF25-75 to predicted ratio** *(%)*	0.89±0.22	0.93±0.22	0.005^*^
**Less than 10 years Working Experience**	**N= 73 (39%)**	**N= 114 (61%)**	
**FEV1/FVC** *(%)*	79.06±5.80	79.56±5.18	0.54
**FVC to predicted ratio** *(%)*	0.94±0.09	0.94±0.11	0.60
**FEV1 to predicted ratio** *(%)*	0.92±0.10	0.92±0.10	0.96
**FEF25-75 to predicted ratio** *(%)*	0.88±0.21	0.90±0.20	0.60
**More than 10 years Working Experience**	**N= 358 (56.6%)**	**N= 275 (43.4%)**	
**FEV1/FVC** *(%)*	78.53±5.49	79.57±4.54	0.005^*^
**FVC to predicted ratio** *(%)*	0.94±0.10	0.96±0.19	0.28
**FEV1 to predicted ratio** *(%)*	0.92±0.11	0.95±0.17	0.008^*^
**FEF25-75 to predicted ratio** *(%)*	0.89±0.23	0.94±0.22	0.003^*^

The painters were classified into two groups, based on their work experience. Almost all spirometric parameters were lower in the exposed group with over ten years of work experience, compared to the non-exposed group. There was a significant difference in terms of the mean predicted values of FEV1/FVC, FEV1, and FEF25-75 between the two groups (P<0.01). Moreover, painters with more than ten years of work experience were subsequently classified according to the exposure type (solvent- or water-based paints). However, considering the intergroup age difference, no significant difference was observed in the predicted values of FVC, FEV1, and FEF25-75 between painters exposed to solvent-based paints and those exposed to water-based paints. The results are shown in Table 2.

**Table 2.** Comparing age, working experience, height and spirometry indexes between solvent based and water based painters with more than 10 years working experience as painter

	**Solvent based Painters N= 193 (53.9%)**	**Water based Painters N= 165 (46.1%)**	**P value**
**Age** (years)	37.72±2.57	37.10±2.39	0.06
**Working Experience** (years)	14.34±1.83	13.96±1.90	0.05
**Height** (centimeter)	176.19±5.90	175.81±6.87	0.57
**FEV1/FVC** (%)	78.55±5.35	78.50±5.66	0.93
**FVC to predicted ratio** (%)	0.93±0.10	0.95±0.11	0.14
**FEV1 to predicted ratio** (%)	0.91±0.11	0.92±0.11	0.26
**FEF to predicted ratio** (%)	0.89±0.23	0.89±0.22	0.85

## DISCUSSION

The present study was conducted on 820 workers to determine the impact of spray painting workshop conditions on the spirometric parameters. The main strength of this study was conducting a survey on a large sample size in one of the largest automobile manufacturing plants in Iran. In this regard, several studies on different spirometric parameters have described significantly lower FVCs in automobile painters, compared to the control group (14–19). However, in the present study, no significant difference was found between the FVCs of the two study groups, which is not consistent with the findings of several studies (14, 16, 17).

Among workers with less than ten years of work experience, spray painters showed better results than the control group, although the difference was not statistically significant. This finding may be accidental or attributed to the recruitment of workers with healthier respiratory systems from the painting workshop and unhealthy workers from other sectors. The present results are in line with a previous study, which found no significant difference in FEV1 and FVC between exposed and non-exposed workers with less than five years of work experience (20). On the other hand, spirometric parameters, including FEV1, FVC, and FEF25-75, were significantly lower among exposed workers with over ten years of work experience, compared to non-exposed workers.

Despite the lack of a significant difference in the mean height, weight, and anthropometric indices of the two groups, a significant difference was observed in the mean age of the exposed and non-exposed groups. Regarding the effect of age and other variables on PFT, the observed changes in FEV1 and FEF25-75 were examined by comparing the measured and predicted values in each group; the obtained results confirmed the variations. Therefore, any difference in the spirometric parameters is only attributable to exposure. Based on the assumption that healthier workers with shorter work experience achieve better results, long-term exposure can result in conflicting outcomes and lower spirometric parameters.

According to previous studies, longer exposure to isocyanides, even at deficient concentrations, reduces the spirometric parameters and results in minimal yet detectable changes in the respiratory tract (11, 21). Although exposure levels were all below the permissible limits in the current study, the spirometric changes, particularly FEF25-75 and FEV1, in workers with long work experience supported our hypothesis.

Moreover, in the present study, painters with more than ten years of work experience were classified according to the type of exposure (solvent- or water-based paints). However, no significant difference was observed in the spirometric parameters of the two groups. This finding is in line with the results of some previous surveys, which indicated the exacerbation of respiratory symptoms following exposure to paints containing more volatile organic compounds (VOCs) than other paints (22, 23). It should be noted that although paint producers have reduced the amount of VOCs in water-based paints, the majority of paints still contain such compounds. Therefore, the use of these paints might have resulted in the lower values of spirometric parameters in workers, compared to the control group unexposed to VOCs.

## CONCLUSION

The findings of this study showed the impact of automobile spray painting on the spirometric parameters. The slight decrease in the mean values of these parameters versus the control group indicated the adequacy of protective measures. According to the current environmental assays, the concentration of paint vapors in the air is at permissible levels due to the use of engineering controls and suitable ventilation. However, the slight decrease in the spirometric parameters may put exposed workers at risk of further pulmonary function impairments. In conclusion, the present results highlighted the necessity of implementing protective measures and routine medical surveillance programs.
